# Supplementary material for: Structural and functional dissection of the DH and PH domains of oncogenic Bcr-Abl tyrosine kinase
Source: Nat Commun. 2017 Dec 13;8:2101. doi: 10.1038/s41467-017-02313-6 (PMC5727386; doi:10.1038/s41467-017-02313-6)
Supplement: Supplementary file 1 — Supplementary Information [file 41467_2017_2313_MOESM1_ESM.pdf]

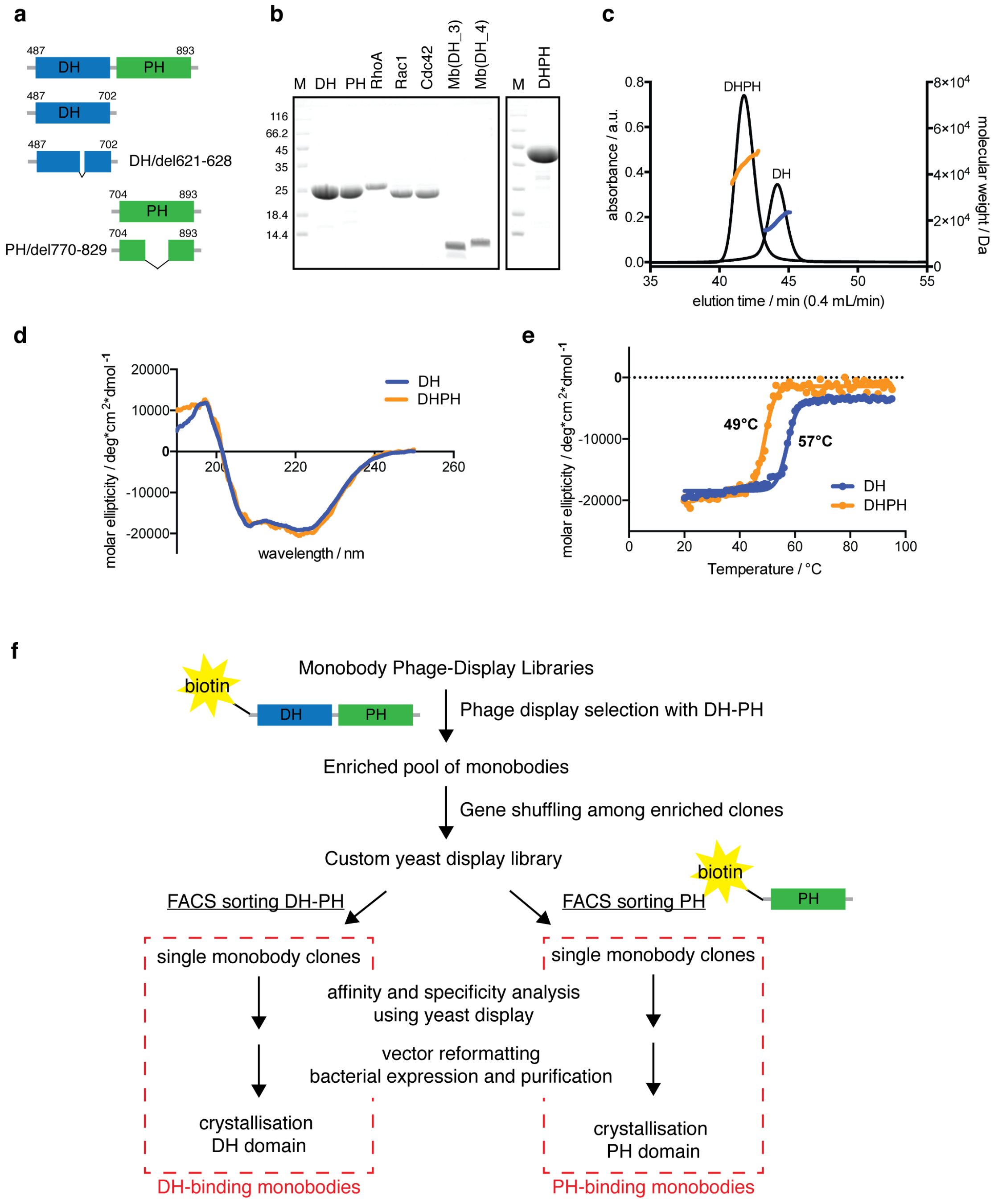

Supplementary Figure 1 Bcr-Abl DH and DH-PH construct design, characterization and monobody screening workflow. (a) Constructs were designed based on domain and secondary structure prediction (prosite.expasy.org, bioinf.cs.ucl.ac.uk/psipred) with reduced unfolded N- and C-terminal linkers. Internal deletion constructs were designed based on the structural studies. (b) Coomassie-stained SDS-PAGE illustrating the purity of the constructs after size exclusion chromatography. (c) SEC-MALS (size exclusion chromatography coupled to multi-angle light scattering) analysis of

the sample homogeneity and oligomeric state. Both constructs, the DH-PH tandem and DH domain, elute in a single peak corresponding to the monomeric size. (d,e) CD spectroscopy for the DH-PH and DH domains. The far-UV spectra of the two constructs (d) are characteristic for a major helical contribution to the secondary structure of the proteins and the melting temperatures recorded at 222 nm (e) indicate well-folded constructs. (f) Using the DH-PH tandem domain conjugated with a biotin via the Avi-tag, we performed four rounds of phage display library selection[1]. We transferred the enriched pool of phage-displayed monobody clones into the yeast display format after applying gene shuffling. Following rounds of FACS sorting of yeast-display libraries, we identified individual monobody clones. We expressed and purified these clones and analyzed their affinity and epitopes. As we found that all of these chosen clones bound to the DH domain but not to the PH domain, we performed additional sorting of the same yeast libraries using the biotinylated PH domain as the target, from which we identified clones binding to the PH domain. The figure is adapted from [2].

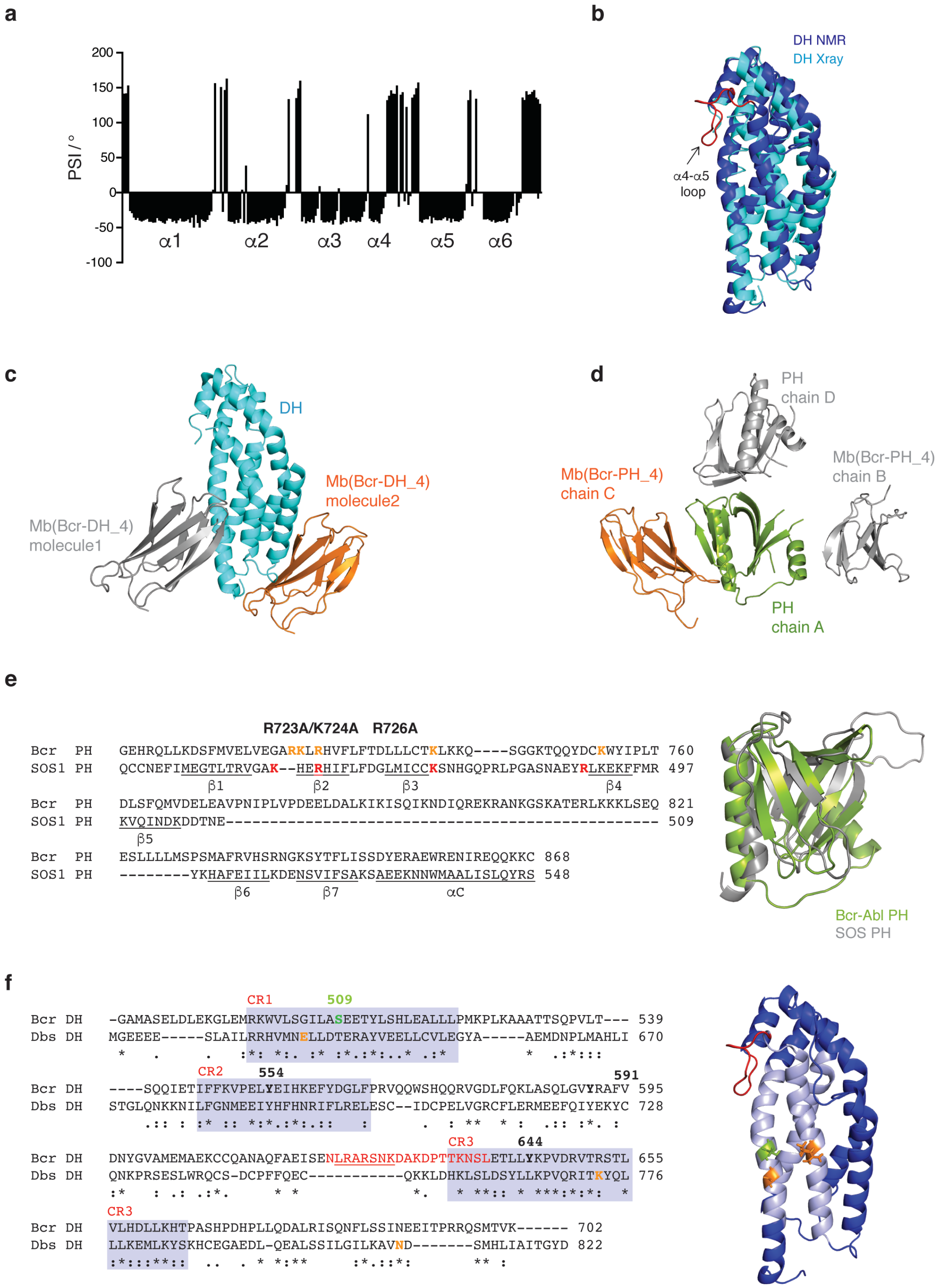

Supplementary Figure 2 Bcr-Abl DH and PH structure determination by NMR and Xray crystallography. (a) Assignment of the six α-helical elements of the DH domain based on the NMR backbone assignment. The psi dihedral angle was obtained by TALOS (prediction of protein backbone torsion angles from NMR chemical shifts, spin.niddk.nih.gov/bax/nmrserver/talos/) and

plotted against the DH domain residues. (b) Overlay of the DH structures obtained by NMR (blue) and X-ray crystallography (cyan). Alignment of the two structures using PyMOL gave a root mean square deviation of 2.0 Å. The major difference between the two structures is the absence of the long α4-α5 loop in the crystallography data and the 14-residue longer C-terminus in the NMR structure. Both elements show a high degree of flexibility in the NMR structure, which explains the missing electron densities. (c) Assignment of the functionally relevant complex of the DH domain and monobody Mb(Bcr-DH_4, related to PDB ID 5N7E). The asymmetric unit of the crystal contains one molecule each of the DH domain and the molecule (DH in cyan and Mb(Bcr-DH_4) molecule 1 in grey). However, the interface between these molecules is not mediated by the residues of the monobody that are diversified in the library. The diversified region of MB(Bcr-DH_4) is limited the FG-loop comprising 11 amino acids (Supplementary Figure 3), which therefore constitutes the binding site to the target. In contrast, there is a second monobody from the neighboring asymmetric unit, that makes direct contact with the DH domain using the FG-loop residues, the Mb(Bcr-DH_4) molecule 2 in orange. Thus, we assigned the DH domain and Mb(Bcr-DH_4) molecule 2 as the biologically relevant assembly. (d) Biologically relevant assembly of the PH domain in complex with monobody Mb(Bcr-PH_4). The asymmetric unit of the crystal comprised two molecules of the PH domain and two molecules of the monobody. Based on biochemical data, we expected a 1:1 complex with the interface formed primarily by the diversified positions of the monobody. The correct biological assembly is therefore represented by the PH domain in chain A (green) and the monobody molecule in chain C (orange), while the other two molecules form complexes with molecules in the neighboring asymmetric units. (e) Structural alignment of the Bcr-Abl PH domain protein sequence to a close homologue, the SOS1 PH domain. The PIP binding residues in SOS1 are highlighted in red and the secondary structure elements are underlined. The PIP binding residues in the Bcr-Abl PH domain can be assigned based on sequence similarity and were further confirmed in the crystal structure. On the right side, overlay of PH domain structures of Bcr-Abl

(green) and SOS1 (grey, PDB ID 1DBH). Both structures agree well in the canonical PH-domain fold comprising the seven-stranded β-sheet and the C-terminal α-helix but vary in the lengths of the connecting loops. (f) Protein sequence alignment of the Bcr-Abl DH domain with the DH domain of Dbs. Overall the sequences have 18% sequence identity with an accumulation in the conserved regions (CR) 1, 2 and 3 delineated by the boxes in light blue. Residues that are critical for GTPase interaction in Dbs are colored in orange. Tyrosine phosphorylation sites of the Bcr-Abl DH domain mapped by mass spectrometry and are highlighted in bold black[3],[4],[5]. S509A, the position of the single point mutation in the Bcr-Abl DH domain that putatively disrupts DH domain function is colored in green[6]. The long α4-α5 loop region of the Bcr-Abl DH domain is indicated in red with the residues that were removed for the internal deletion mutant underlined. On the right side, the Bcr-Abl DH domain structure with CR1 – CR3 in light blue and the α4-α5 loop in red is shown. G505 and R652, residues aligning to those important for Dbs-GTPase interaction in CR1 and CR2 (E639 and K774), are in orange stick representation and S509 in green.

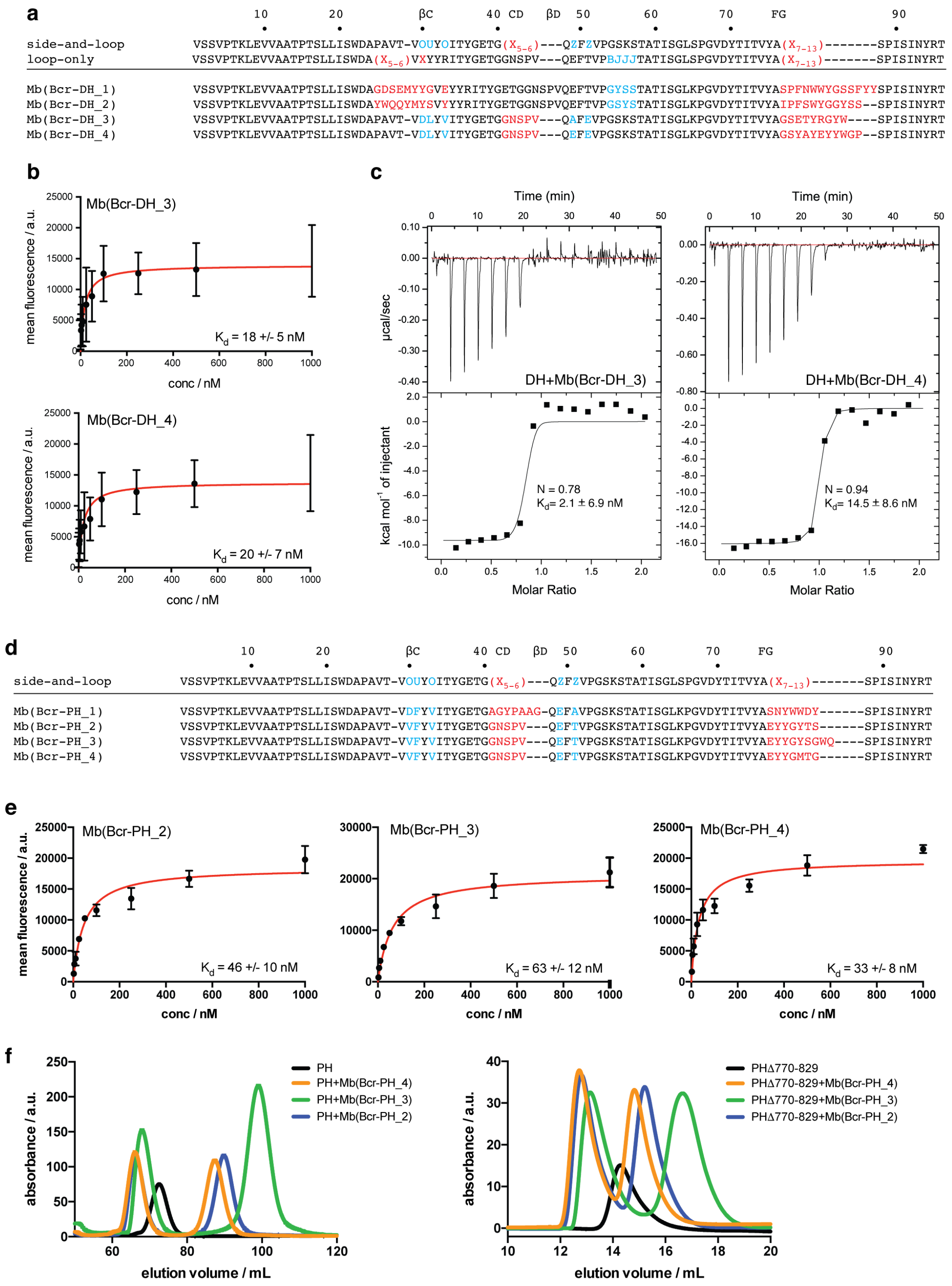

Supplementary Figure 3 Characterization of DH- and PH-binding monobodies. (a) The sequence of the four DH-binding monobodies is shown together with the two different libraries on top. Diversified regions are color-coded. "X" denotes a mixture of 30% Tyr, 15% Ser, 10% Gly, 5% Phe, 5% Trp, and 2.5% each of all the other amino acids except for Cys; "B", a mixture

of Gly, Ser, and Tyr; “J”, a mixture of Ser and Tyr; “O”, a mixture of Asn, Asp, His, Ile, Leu, Phe, Tyr, and Val; “U”, a mixture of His, Leu, Phe, and Tyr; “Z”, a mixture of Ala, Glu, Lys, and Thr, as described previously[1]. (b) Binding of monobody clones Mb(Bcr-DH_3) and Mb(Bcr-DH_4) displayed on yeast surface to the biotinylated DH-PH domain. The curves show the best fit of a 1:1 binding mode to the data (Prism, GraphPad). Each point for the yeast binding assay corresponds to the average of two repeats +/- SD. These binding experiments were repeated twice and a representative example is shown. Both clones bind with low nanomolar affinity to the DH-PH tandem domain. (c) ITC (isothermal titration calorimetry) measurements for monobody clones Mb(Bcr-DH_3) and Mb(Bcr-DH_4) titrated to the DH domain. The titrations were performed at 25 °C with 20 µM DH and 200 µM monobody stock solutions. The top panels show the raw heat signal of an ITC experiment. The bottom panels show the integrated calorimetric data of the area of each peak with the continuous line representing the best fit of the data based on a 1:1 binding model (MicroCal software). (d) The sequence of the four PH-binding monobodies is shown together with the side-and-loop library on top. The library designs are described in (a). (e) Yeast binding assay for monobody clones Mb(Bcr-PH_2), Mb(Bcr-PH_3) and Mb(Bcr-PH_4) using biotinylated PH. Data points were fitted assuming a 1:1 binding mode in Prism (GraphPad). Each point for the yeast binding assay corresponds to the average of two repeats +/- SD. These binding experiments were repeated twice and a representative example is shown. All three clones bind with low affinity to the PH domain. (f) Size exclusion chromatography of the full-length PH domain and PHΔ770-829 in complex with the monobody clones Mb(Bcr-PH_2), Mb(Bcr-PH_3) and Mb(Bcr-PH_4). The PH and monobody samples were mixed prior to injection to the column in a 1:2 molar ratio (PH:monobody). Thus, in the elution profiles, the first peak corresponds to the PH or PH-monobody complex and the second peak corresponds to free monobody. The complexes involving the full-length PH domain were analyzed on a Superdex 75 16/600 column and samples for the PHΔ770-829 were run on a Superdex 75 10/300.

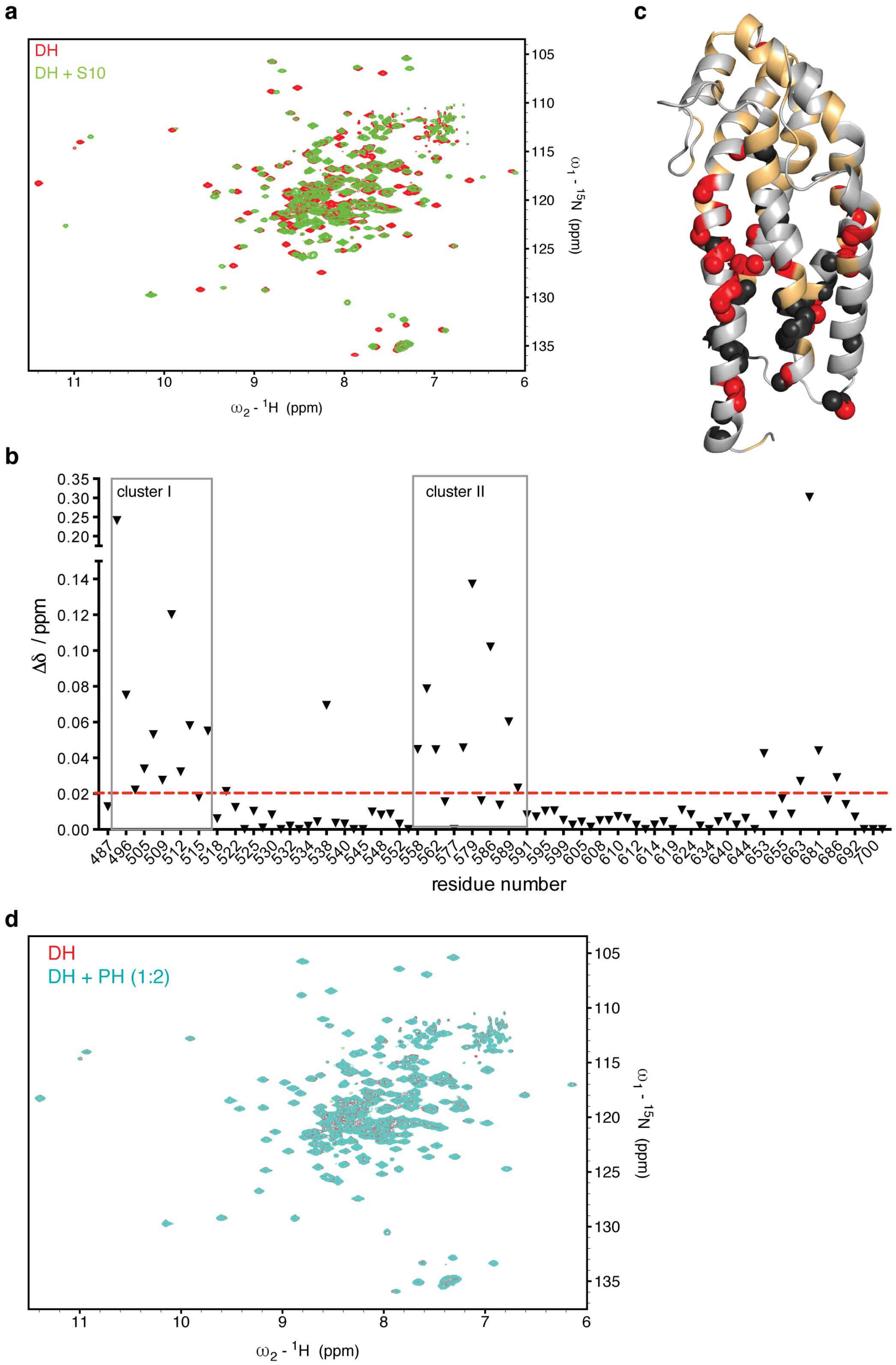

Supplementary Figure 4 NMR interaction studies of the DH domain with Mb(Bcr-DH_4) and the PH domain. (a) NMR chemical shift changes upon binding of Mb(Bcr-DH_4) to the DH domain. Overlay of [^{15}N,^{1}H]-TROSY

experiments for the DH domain alone (red) or in complex with the Mb(Bcr-DH_4) monobody (green). (b) Chemical shift perturbations (CSPs) upon addition of the monobody for all resolved and assigned peaks in the [^{15}N,^{1}H]-TROSY. The composite ^{15}N and ^{1}H chemical shift perturbation (Δδ) was calculated with a scaling factor for the ^{15}N shift changes of 0.14. Due to the large changes in the spectrum of the DH/ Mb(Bcr-DH_4) complex, the assignment for 13% of the resonances could not be transferred. Also overlapping resonances were excluded from the analysis. Most CSPs are observed in two clusters corresponding to residues in helices 1,2 and 3. The red line in the graph indicates the threshold used for (c). (c) Chemical shift perturbations mapped on the DH structure. All residues with CSPs above the threshold of 0.02 ppm for the ^{15}N and ^{1}H composite chemical shift changes (Δδ) were colored in red, the amide nitrogen of these residues are represented as spheres for better visibility. Black residues could not be assigned in the DH/ Mb(Bcr-DH_4) complex due to large CSPs or line broadening, gray residues were not included in the analysis due to peak overlap and beige colored residues remained unaffected. Since binding of the monobody caused large perturbations in the DH domain spectrum, precise definition of the monobody binding epitope is not possible based on this experiment. (d) Overlay of [^{15}N,^{1}H]-TROSY spectra for the ^{15}N-labeled DH domain at 150 µM alone (red) and in a 1:2 molar mixture with the PH domain (DH:PH). Spectra look identical with minimal chemical shift changes indicating that the DH and PH domain do not interact when in *trans*.

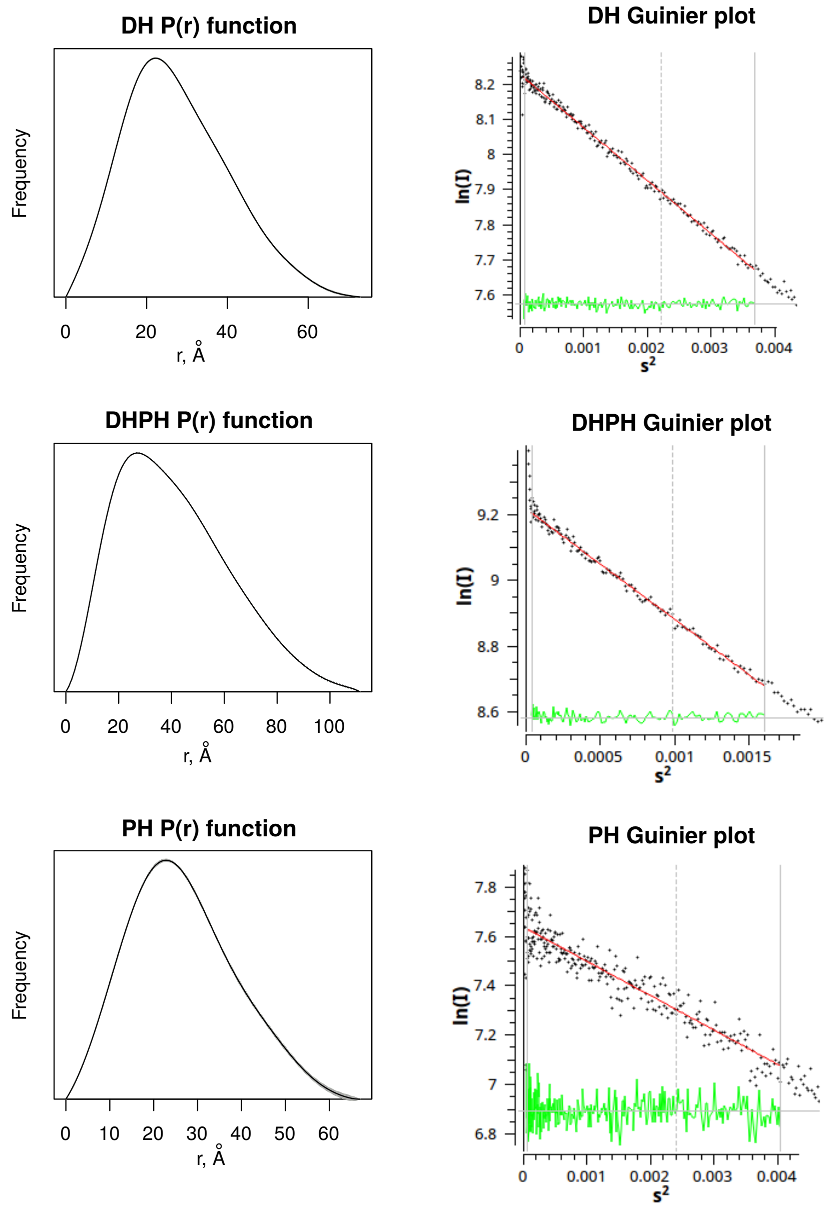

Supplementary Figure 5 Small-angle X-ray scattering (SAXS) of the DH-PH tandem domain. Plots of P(r) functions and Guinier plots of the SAXS data of the DH-, PH- and DH-PH domains.

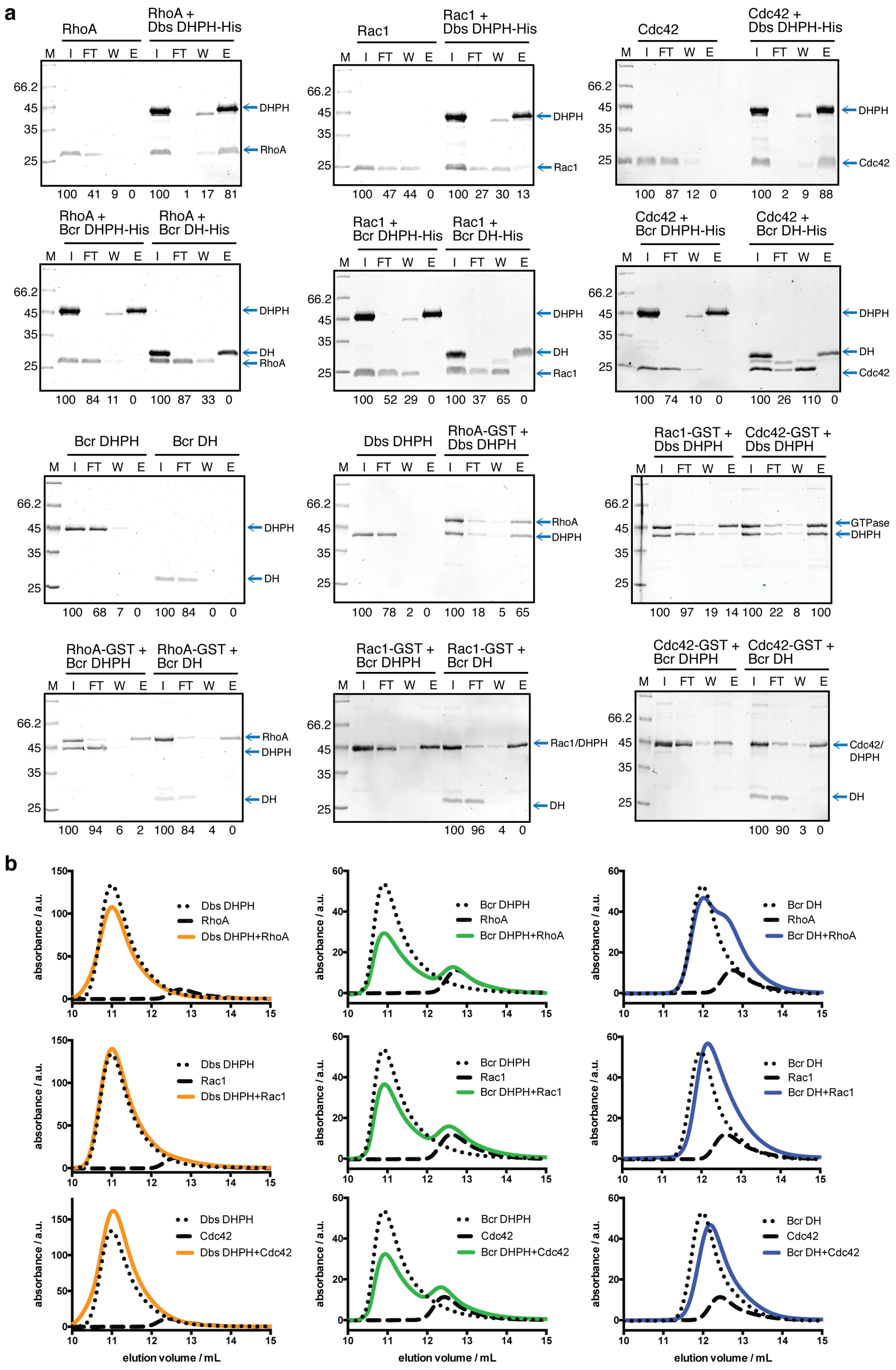
a
RhoA
RhoA + Dbs DHPH-His
M I FT W E I FT W E
66.2
45
35
25
DHPH
RhoA
100 41 9 0 100 1 17 81
Rac1
Rac1 + Dbs DHPH-His
Rac1
100 47 44 0 100 27 30 13
Cdc42
Cdc42 + Dbs DHPH-His
Cdc42
100 87 12 0 100 2 9 88
RhoA + Bcr DHPH-His
RhoA + Bcr DH-His
DH
100 84 11 0 100 87 33 0
Rac1 + Bcr DHPH-His
Rac1 + Bcr DH-His
100 52 29 0 100 37 65 0
Cdc42 + Bcr DHPH-His
Cdc42 + Bcr DH-His
100 74 10 0 100 26 110 0
Bcr DHPH
Bcr DH
100 68 7 0 100 84 0 0
Dbs DHPH
RhoA-GST + Dbs DHPH
100 78 2 0 100 18 5 65
Rac1-GST + Dbs DHPH
Cdc42-GST + Dbs DHPH
GTPase
100 97 19 14 100 22 8 100
RhoA-GST + Bcr DHPH
RhoA-GST + Bcr DH
100 94 6 2 100 84 4 0
Rac1-GST + Bcr DHPH
Rac1-GST + Bcr DH
Rac1/DHPH
100 96 4 0
Cdc42-GST + Bcr DHPH
Cdc42-GST + Bcr DH
Cdc42/ DHPH
100 90 3 0
b
absorbance / a.u.
elution volume / mL
Dbs DHPH
RhoA
Dbs DHPH+RhoA
Bcr DHPH
Bcr DHPH+RhoA
Bcr DH
Bcr DH+RhoA
Dbs DHPH+Rac1
Bcr DHPH+Rac1
Bcr DH+Rac1
Dbs DHPH+Cdc42
Bcr DHPH+Cdc42
Bcr DH+Cdc42

Supplementary Figure 6 Interaction studies of the Bcr-Abl DH-PH and DH domains with Rho GTPases RhoA, Rac1, Cdc42. (a) RhoA, Rac1 and Cdc42 are purified with and without an N-terminal GST-tag and the DH/DH-PH domain constructs are purified with and without N-terminal His-tag. Pull-down experiments are either conducted with His-tagged DH/DH-PH constructs plus unlabeled GTPase on Ni-Sepharose beads or with GST-tagged GTPases plus untagged DH/DH-PH constructs using glutathione sepharose beads. Equal amounts of input, flow-through, wash and elution fractions were analyzed on SDS-PAGE, scanned with the Odyssey® imager (Li-Cor) and quantified using the Image Studio Software (Li-Cor). The percentage of untagged protein in the respective gel lane is indicated below the gel normalized to the input fraction. The pull-down of the DH-PH domain with GST-tagged Rac1 and Cdc42 could not be quantified due to overlapping bands. (b) Overlay of size exclusion chromatography profiles for the DH/DH-PH constructs alone (dotted line), GTPase alone (dashed line) and after mixing the two proteins (colored line). In the upper panel, the Dbs DH-PH sample elutes in a single peak together with RhoA (orange), while the Bcr-Abl constructs (blue/green) and RhoA elute in separate peaks and thus do not interact in this experiment. Similar results were obtained also for Rac1 (middle panel) and Cdc42 (lower panel).

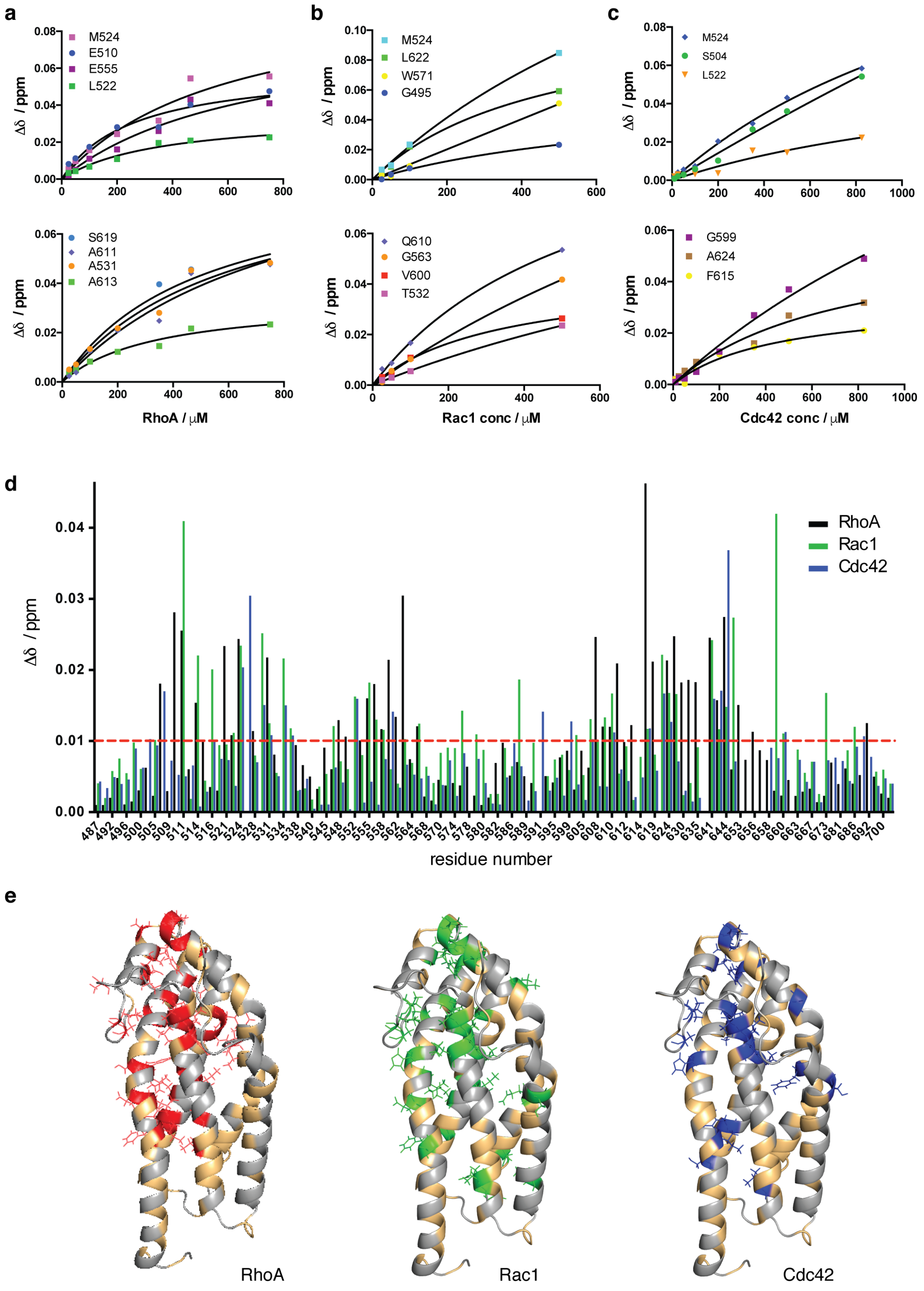

Supplementary Figure 7 Interaction studies of the Bcr-Abl DH domain with Rho GTPases RhoA, Rac1, Cdc42 by NMR spectroscopy. (a) NMR titration of ^{15}N-labeled DH domain with RhoA. The chemical shift perturbation for representative resonances of the DH domain upon addition of RhoA is plotted

against the GTPase concentration. Data points are fitted assuming a 1:1 binding mode in Prism (GraphPad) to derive the K_d value. Individual values can be interpreted as the lower threshold for the DH-GTPase interaction clustering around a mean K_d value +/- s.d. (b) NMR titration of ^{15}N-labeled DH domain with Rac1. Data points are processed as in (a). (c) NMR titration of ^{15}N-labeled DH domain with Cdc42. Data points are processed as in (a). (d) Chemical shift perturbations upon addition of the GTPases for all resolved and assigned peaks in the [^{15}N,^{1}H]-TROSY. The composite ^{15}N and ^{1}H chemical shift perturbation (Δδ) was calculated with a scaling factor for the ^{15}N shift changes of 0.14. (e) Mapping of chemical shift perturbations (CSPs) larger than 0.01 ppm on the structure of the DH domain with red for RhoA-induced CSPs, green fro Rac-induced CSPs and blue for Cdc42-induced CSPs. Side chains of these residues are represented as lines for better visibility. Residues colored in grey were not considered due to overlap in the [^{15}N,^{1}H]-TROSY, residues in beige remained unaffected. All three GTPases induce a very similar pattern of chemical shit changes accumulating around the seat-back region of the domain also involving the α4- α5 loop.

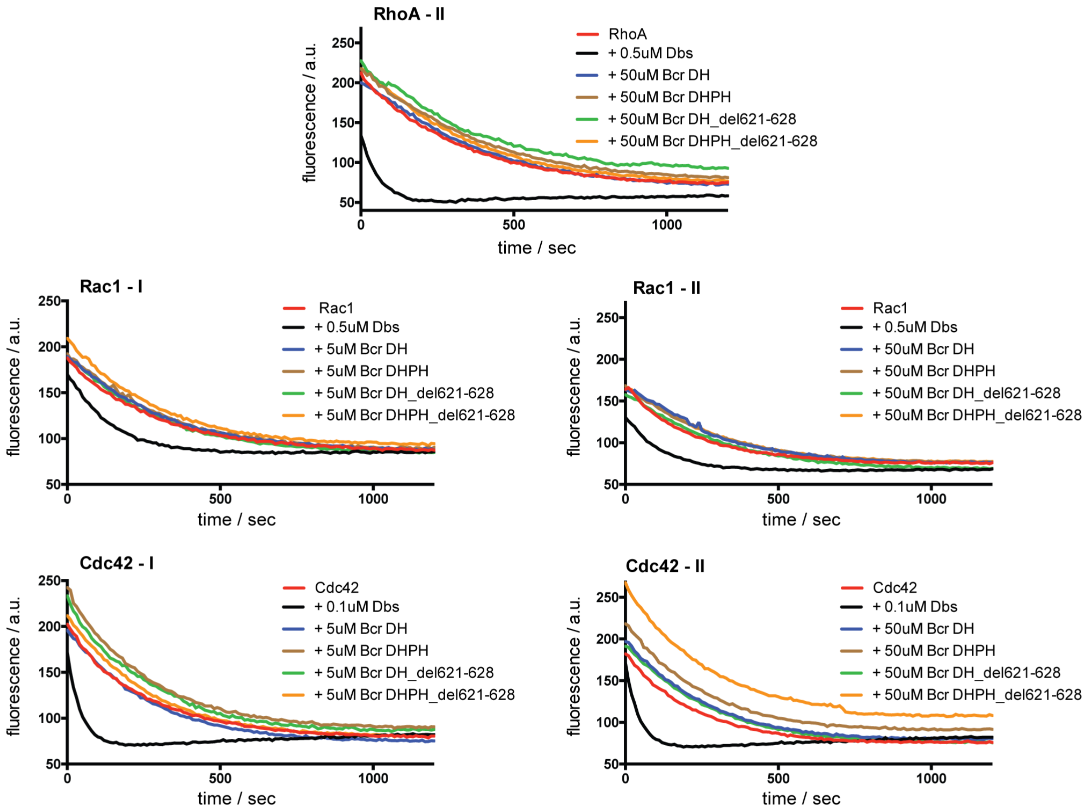

Supplementary Figure 8 Rho GTPase nucleotide exchange. Nucleotide exchange rates for Rho GTPases in the presence of DH and DH-PH constructs. The rate of nucleotide exchange was monitored in the presence of different DH-PH constructs using Dbs DH-PH as a positive control. Experiments were done in duplicates and at two different concentrations of the Bcr-Abl DH/DH-PH constructs for RhoA (upper panel), Rac1 (middle) and Cdc42 (lower panel).

a

R723A/K724A R726A

```
Bcr  PH     GEHRQLLKDSFMVELVEGARKLRHVFLFTDLLLCTKLKKQ----SGGKTQQYDCKWYIPLT 760
SOS1 PH     QCCNEFIMEGTLTRVGAK--HERHIFLFDGLMICCKSNHGQPRLPGASNAEYRLKEKFFMR 497
                      β1           β2          β3                     β4
Bcr  PH     DLSFQMVDELEAVPNIPLVPDEELDALKIKISQIKNDIQREKRANKGSKATERLKKKLSEQ 821
SOS1 PH     KVQINDKDDTNE------------------------------------------------- 509
               β5
Bcr  PH     ESLLLLMSPSMAFRVHSRNGKSYTFLISSDYERAEWRENIREQQKKC 868
SOS1 PH     --------YKHAFEIILKDENSVIFSAKSAEEKNNWMAALISLQYRS 548
                       β6         β7             αC
```

b

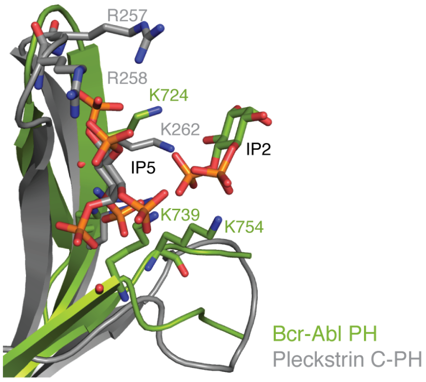

c

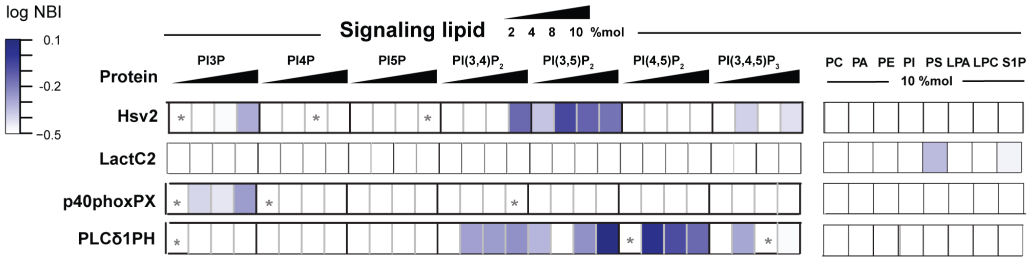

d

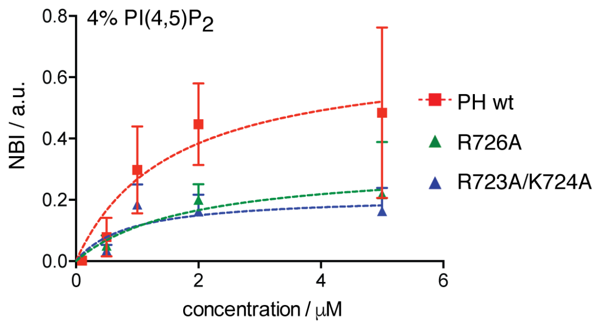

ID liposome	Kd (µM)
PC_PI(3,4)P_2_4	4.2
PC_PI(3,5)P_2_4	0.7
PC_PI(4,5)P_2_4	1.6
PC_PI(3,4,5)P_3_4	7.6

Supplementary Figure 9 PIP binding of the PH domain. (a) HHpred sequence alignment of the Bcr-Abl PH domain protein sequence to a close homologue, the human SOS1 PH domain. The PIP binding residues in SOS1 are highlighted in red and the secondary structure elements are underlined. The PIP-binding residues in the Bcr-Abl PH domain were assigned here

based on sequence similarity and were then further confirmed in the crystal structure. (b) Comparison of the PIP binding site of the Pleckstrin PH-IP5 complex (PDB ID 2I5C) in grey and the Bcr-Abl PH-IP2 complex in green. The ligands are represented in sticks conformation and colored by element. (c) Binding of control proteins to surrogates of biological membranes in the LiMA assay. Domains and proteins with known binding preferences were used as positive control. Hsv2 preferentially binds to liposomes containing PI3P and PI(3,5)P_2, LactC2 to liposomes containing PS, p40phoxPX to liposomes containing PI3P and PLCδ1PH to liposomes containing PI(4,5)P_2. PI(3)P, PI(4)P, PI(5)P, PI(3,4)P_2, PI(3,5)P_2, PI(4,5)P_2 and PI(3,4,5)P_3 are phosphoinositides. Abbreviations are Lact, lactadherin; PC, phosphatidylcholine; PA, phosphatidic acid; PE, phosphatidylethanolamine; PI, phosphatidylinositol; PS, phosphatidylserine; LPA, lyso PA; LPC, lyso PC; S1P, sphingosine-1-phosphate; NBI, normalized binding intensity. (*) indicates not determined values. Values are means (n = 3). (d) PH domain lipid-binding affinity. Dissociation constants (K_d) were derived from titration experiments using PC liposomes containing 4% of the respective PIPs in LiMA. K_d values were determined assuming a 1:1 binding mode in Prism (GraphPad). Values to derive the lipid binding affinity were taken from Fig. 5D and represent averages of three repeats +/- SD.

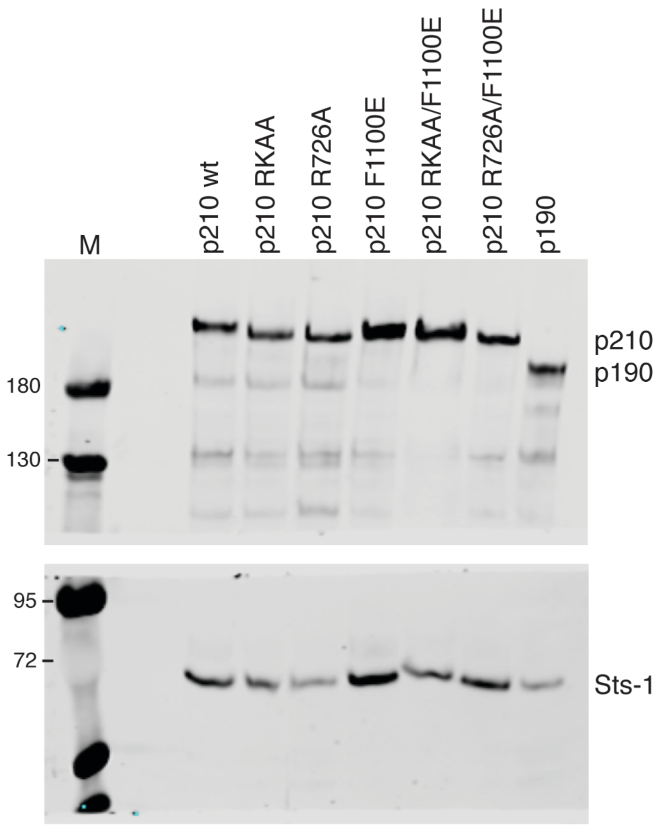

Supplementary Figure 10 Uncropped immunoblot scans corresponding to Fig. 6c.

Supplementary Table 1 NMR statistics for the Bcr-Abl DH structure calculation (PDB ID 5NR6, BMRB entry 34101).

	DH
**Structural restraints**	
Assigned NOE distance restraints	4298 (100%)
Short range, $\|i - j\| \leq 1$	2111 (49.1%)
Medium range, $1 < \|i - j\| < 5$	1342 (31.2%)
Long range, $\|i - j\| \geq 5$	845 (19.7%)
Dihedral angle restraints (φ/ψ)	358
**Structure statistics**	
Average CYANA target function value ($Å^2$)	3.88 ± 0.10
Average AMBER energy (kcal/mol)	-8774 ± 217
**Restraint violations**	
Maximal distance restraint violation (Å)	0.28
Number of violated distance restraints > 0.2 Å	1
Maximal dihedral angle restraint violations (°)	2.40
Number of violated dihedral angle restraints > 5°	0
**Ramachandran plot**	
Residues in most favored regions	89.9%
Residues in additionally allowed regions	9.0%
Residues in generously allowed regions	0.8%
Residues in disallowed regions	0.4%
**RMSD (residues 496–619, 638–693)**	
Average backbone RMSD to mean (Å)	0.85 ± 0.15
Average heavy atom RMSD to mean (Å)	1.20 ± 0.14

Supplementary Table 2 Data collection and refinement for the crystal structures of the DH (PDB ID 5N7E) and PH domain (PDB ID 5OCE).

	**DH/Mb(Bcr-DH_4)**	**PH/Mb(Bcr-PH_4)**
**Data collection**		
Space group	C 1 2 1	P 1
Cell dimensions		
a, b, c (Å)	164.12, 55.57, 45.03	29.68 62.61 67.38
α, β, γ (°)	90.00, 105.46, 90.00	62.74 84.77 89.29
Resolution (Å)	43.40-1.65 (1.71-1.65)	33.68-1.65 (1.711-1.65)
R_{merge}	0.04 (0.59)	0.03 (0.63)
$I/\sigma I$	18.30 (1.94)	23.03 (2.07)
Completeness (%)	99.30 (93.30)	97.13 (96.10)
Redundancy	3.8 (3.7)	4.9 (4.6)
**Refinement**		
Resolution (Å)	1.65	1.65
No. of reflections	47054 (4391)	49948 (4899)
R_{work}/R_{free}	0.18/0.22	0.17/0.20
No. of atoms		
Protein	2254	3284
Ligand/ion	-	62
Water	348	253
B-factors		
Protein	34.97	39.40
Ligand/ion	-	67.83
Water	43.70	44.65
rmsd		
Bond lengths (Å)	0.006	0.011
Bond angles (°)	0.81	1.07

Supplementary Table 3 Data collection and structural parameters obtained by SAXS.

	DH	DH-PH	PH
**Data collection parameters**			
Instrument	P12	P12	P12
Wavelength (Å)	1.24	1.24	1.24
s-range $(Å)^{-1}$	0.0025-0.48	0.0025-0.48	0.0027-0.45
Exposure time (s)	1	1	1
Concentration range (mg/mL)	1.8-14.4	1.8-14.4	1.8-9.1
Temperature (K)	293	293	283
**Structural parameters**			
R_g (Å) [from *P(r)*]	22 ±1	32 ±2	21 ±2
R_g (Å) (from Guinier)	21 ±2	32 ±2	20 ±2
D_{max} (Å)	73 ±5	111 ±10	66 ±5
Porod volume ($Å^3$ x 10^3)	38 ±2	69 ±5	38 ±3
**Mol. mass determination (kDa)**			
From *I(0)*	25 ±2	41 ±4	16 ±2
From Porod volume	23 ±2	42 ±4	23 ±2
From AA sequence	24.8	47.1	22.4

Data collection and structural parameters obtained by SAXS for the Bcr-Abl DH (SASDC26), PH (SASDC36) and DH-PH domains (SASDC46). Molecular mass (M) was estimated from forward scattering I(0) and Porod volume respectively. Radius of gyration, R_g (Å) was calculated using the Guinier approximation and also the distance distribution function (*P*(*r*) using GNOM), which also estimates maximum particle dimension (D_{max}).

Supplementary References

1. Koide, A., Wojcik, J., Gilbreth, R.N., Hoey, R.J. & Koide, S. Teaching an old scaffold new tricks: monobodies constructed using alternative surfaces of the FN3 scaffold. *J. Mol. Biol.* **415**, 393-405 (2012).
2. Stockbridge, R.B. *et al.* Crystal structures of a double-barrelled fluoride ion channel. *Nature* **525**, 548-51 (2015).
3. Goss, V.L. *et al.* A common phosphotyrosine signature for the Bcr-Abl kinase. *Blood* **107**, 4888-97 (2006).
4. Preisinger, C. *et al.* Imatinib-dependent tyrosine phosphorylation profiling of Bcr-Abl-positive chronic myeloid leukemia cells. *Leukemia* **27**, 743-6 (2013).
5. Reckel, S. *et al.* Differential signaling networks of Bcr-Abl p210 and p190 kinases in leukemia cells defined by functional proteomics. *Leukemia* **31**, 1502-1512 (2017).
6. Tala, I. *et al.* Contributions of the RhoGEF activity of p210 BCR/ABL to disease progression. *Leukemia* **27**, 1080-9 (2013).
