# Supplementary material for: Structural and functional dissection of the DH and PH domains of oncogenic Bcr-Abl tyrosine kinase
Source: Nat Commun. 2017 Dec 13;8:2101. doi: 10.1038/s41467-017-02313-6 (PMC5727386; doi:10.1038/s41467-017-02313-6)
Supplement: Supplementary file 3 — Description of Additional Supplementary Files [file 41467_2017_2313_MOESM3_ESM.pdf]

Description of Supplementary Files

File Name: Supplementary Data 1
Description: Tandem-affinity purification mass spectrometry results of the Bcr-Abl DH-PH from K562 cells. Unique peptide counts of proteins found after tandem affinity purification of Strep-HA-tagged DHPH and negative controls (GFP and Shc-1).

File Name: Supplementary Data 2
Description: Liposome composition for the 36 lipid mixtures that were used for the Liposome MicroArray (LiMA) assay.

File Name: Supplementary Data 3
Description: Raw data of the Liposone MicroArray (LiMA) assays shown in Fig. 5d and Supplementary Figure 9c/d.

File Name: Supplementary Data 4
Description: Sequences of the cloning and mutagenesis primers that were used for this study.
